# ASD with ADHD vs. ASD and ADHD alone: a study of the QbTest performance and single-dose methylphenidate responding in children and adolescents

**DOI:** 10.1186/s12888-022-03878-3

**Published:** 2022-04-20

**Authors:** Dejan Stevanovic, Elisabet Wentz, Salmir Nasic, Rajna Knez

**Affiliations:** 1 Gillberg Neuropsychiatry Centre, Institute of Neuroscience and Physiology, Sahlgrenska Academy, University of Gothenburg, Box 430, 40530 Göteborg, Sweden; 2 Psychiatry Department, Clinic for Neurology and Psychiatry for Children and Youth, Dr Subotic 6a, 11000 Belgrade, Serbia; 3 Institute of Neuroscience and Physiology, Sahlgrenska Academy, University of Gothenburg, Box 430, 40530 Göteborg, Sweden; 4 Research & Development Centre, Skaraborgs Hospital, Lövängsvägen, 541 42 Skövde, Sweden; 5 Department of Molecular and Clinical Medicine, Institute of Medicine, Sahlgrenska Academy, University of Gothenburg, Box 430, 40530 Göteborg, Sweden; 6 Department of Pediatrics, Skaraborgs Hospital, Skövde, Sweden

**Keywords:** Continuous performance task, Inattention, Impulsivity, Motor activity, Stimulants, Autism

## Abstract

**Background:**

The continuous performance task (CPT) may help identify coexistent attention deficit hyperactivity disorder (ADHD) in autism spectrum disorder (ASD). The Quantified behavior Test (QbTest) combines a CPT and motion-tracking data to assess ADHD symptoms. This study aimed to evaluate the QbTest performance of children and adolescents with ASD plus ADHD, including estimating the effects of single-dose methylphenidate (MPH). To achieve these aims, (1) the QbTest performances were evaluated in ASD alone, ASD plus ADHD, and ADHD alone, and (2) the effects on the QbTest performance of single-dose MPH before and after intake were estimated across the groups. It was assumed that the ASD plus ADHD performance, including the MPH response, would preferably resemble the performance in ADHD alone, rather than ASD alone.

**Methods:**

Retrospective data were analyzed for 482 children and adolescents: 69 with ASD alone, 142 with ASD plus ADHD (ASD/ADHD), and 271 with ADHD alone. For 343 subjects, the QbTest was performed before and up to four hours after a single-dose MPH intake. A summary index of the CPT and motion-capture data was provided for QbTest cardinal parameters.

**Results:**

Of 12 QbTest parameters assessed before given MPH, the ASD/ADHD group had scores in line with the ASD group regarding four parameters and the ADHD group regarding nine parameters. Significant differences between groups were seen with respect to QbInattention (*p* > 0.05); the lowest scores in ASD and the highest in ADHD. Those with ASD/ADHD and ADHD had similar QbActivity and QbImpulsivity scores, but significantly higher than those with ASD. After MPH intake, scores for QbActivity decreased similarly in ASD/ADHD and ADHD, as well as scores for QbImpulsivity. QbImpulsivity increased in ASD. QbInattention scores decreased similarly in all groups after MPH intake.

**Conclusions:**

Children and adolescents with ASD plus ADHD exhibited more atypical QbTest performances than those with ASD alone, while most of their performances were similar to those observed in ADHD alone. In addition, a single dose of MPH mitigated attention deficits and decreased hyperactivity while improved impulsivity in these children. Prospective studies should further clarify the role of the QbTest in the diagnostic and therapeutic interventions in ASD with ADHD.

## Background

Autism spectrum disorder (ASD) and attention deficit hyperactivity disorder (ADHD) are distinguished neurodevelopmental conditions in the current classification systems [1, 2]. Both conditions are characterized by different clinical presentations of core symptoms and a heterogeneous spectrum of various co-occurring psychiatric and medical morbidities. The etiologies are multifactorial, including the complexity of genetic backgrounds, while neurocognitive differences are ubiquitous (e.g., [3–7]).

Over the past two decades, epidemiological, clinical, genetic, neuropsychological, and neuroimaging studies have generated increasing evidence for commonalities between ASD and ADHD [8–10]. In 2010 when the Early Symptomatic Syndromes Eliciting Neurodevelopmental Clinical Examinations (ESSENCE) was launched, as an umbrella term that implies the coexistence of neurodevelopmental disorders from an early age, it was made an important leap in understanding commonalities between ASD and ADHD and their mutual expression of symptoms [11]. As of 2013, when the latest edition of the Diagnostic and Statistical Manual of Mental Disorders (DSM-5) [1] was launched, an individual can be assigned ASD and ADHD concurrently if criteria for both disorders are met. According to two recent epidemiological meta-analyses, the proportion of children and adolescents with ADHD who meet the criteria for ASD across studies ranges from 12 to 40% (pooled prevalence of 21%) [12], and between 2 and 86% (pooled prevalence of 28%) of those with ASD also fulfill the ADHD criteria depending on samples studied [13]. The clinical observation of a high phenotypical overlap between ASD and ADHD suggests the possibility of the coexistence of ADHD and ASD within children (e.g., [14]); for example, children with both ADHD and ASD more often belong to the ADHD subtype with combined presentation than children with ADHD only [15]. Moreover, comorbidity with ADHD may constitute a distinctive phenotype of ASD, with more severe symptoms, more adaptive problems, and more significant academic dysfunctions [16].

Objective measures for assessing neurocognitive aspects, like sustained and selective attention, information processing, abstract concept formation, or complex problem-solving abilities, may play a role in increasing diagnostic accuracy and optimizing treatment response in ADHD (e.g., [17, 18]). In this regard, they may minimize or eliminate the effects of a heterogeneous spectrum of various co-occurring psychiatric morbidities. As of recently, findings from studies measuring neurocognitive aspects have also generated valuable data for understanding ASD and/or ADHD and have indicated that neurocognitive assessments, such as continuous performance tasks (CPTs) with or without motion analysis, along with standard clinical rating scales, may yield the correct diagnostic classification of the two conditions (e.g., [19, 20]). In addition, data on neurocognitive aspects indicate overlapping and unique patterns of cognitive impairments in children with ASD compared with children with ADHD [21]. At the same time, those with a combination of ASD and ADHD may have an additive co-occurrence of attention and inhibition deficits of both disorders independently [22, 23]. Although no firm evidence exists, it is indicative that CPTs measuring sustained/selective attention and impulsivity may have adjuvant roles in diagnosing and managing ADHD (e.g., [18, 24]). In addition, CPTs may contribute when classifying the combination of ASD and ADHD compared with ASD and ADHD alone [25], or may contribute to detecting attentional changes in ASD [26, 27].

Over the past 15 years, evidence has emerged for the effects of stimulant medications on CPT performance in children with ADHD (e.g., [28–33]. In this regard, CPTs may have a role when identifying children with ADHD that could respond to methylphenidate (MPH). CPTs could constitute a method for early detection of treatment responders [31], which in turn could be used for determining treatment effects in children with ASD and co-occurring ADHD symptoms [34]. For example, combining Functional Near-Infrared Spectroscopy (fNIRS) data and go/no-go data, Tokuda et al. [35] found that children with ADHD and ASD had different neuro-activation patterns than children with ADHD but without ASD in the right inferior and middle frontal gyri after receiving a single dose of MPH. In addition, Pearson et al. [36] studied the effects of doses of combined extended-release MPH (ER-MPH) with immediate-release MPH (IR-MPH) on a CPT performance in children with ASD and ADHD. They found that sustained attention and selective attention improved with MPH treatment, as did the potential to inhibit impulsive responses. Another study showed that processing speed, measured by a CPT in children with high-functioning ASD and ADHD, improved after a single dose of MPH, while the quality of any other parameters was not affected [37].

The Quantified behavior Test (QbTest) [38] is a commercially available measure (Qbtech AB, www.qbtech.com) approved by the United States Food and Drug Administration (FDA) to supplement the standard clinical assessment and evaluation of treatment interventions in ADHD (e.g., [18, 39–41]). The QbTest is a frequently utilized CPT in pediatric populations, including ASD (e.g., [40, 42]), although it is not specifically designed to identify or diagnose ASD or monitor its treatment outcome. In addition, the motor activity analysis of the QbTest targets hyperactivity as a proposed ADHD phenotype [43]. However, one study published in 2021 has shown that the QbTest could not distinguish between ADHD and other neurodevelopmental conditions [44].

As part of a series of retrospective studies evaluating QbTest performances in different pediatric populations [33], this study aimed to evaluate the performance of children and adolescents with ASD plus ADHD on the QbTest, including evaluating the effects of single-dose methylphenidate (MPH). To achieve these aims, (1) the QbTest performances among children and adolescents with ASD plus ADHD were compared with the results among those with ASD and ADHD alone, and (2) the effects on the QbTest performance of single-dose MPH were evaluated before and after intake across the groups. It was assumed that the performances in ASD plus ADHD, including MPH response, would resemble the performances in ADHD alone, rather than ASD alone.

## Materials and methods

### Participants and procedures

The current study involved a retrospective chart review of the data of children and adolescents (hereafter referred to as *children* unless otherwise specified) referred at a child and adolescent psychiatry clinic in one of a few general hospitals located in western Sweden during the period 1 January 2004 to 31 December 2017. All children with a suspected neurodevelopmental disorder presented at the clinic went through a diagnostic process according to the clinic’s standard diagnostic procedure. Although data were not available, it was likely that all children referred to the clinic due to some ADHD symptoms were assessed with the QbTest, irrespective of later being assigned a neurodevelopmental, behavioral, or mental disorder diagnosis. In addition, children with ASD were likely suspected or there were reports of attention deficits, irrespective of the diagnosis of ADHD, considering that those with ASD who received MPH had more atypical QbTest scores than those who did not (see below).

Data for this study were extracted from a database containing information on the QbTest, demographic, and selected clinical data. The main inclusion criteria were children aged six to 18 years who underwent a QbTest assessment, and with a diagnosis of ASD, ASD with co-occurring ADHD, or ADHD. Children with the diagnoses of severe mental illness, such as early-onset psychosis, and intellectual disability were not included, nor were children with other co-occurring specific psychiatric diagnoses, such as anxiety, depression, and/or behavioral disorders, nor children with severe speech/language, learning, and/or motor difficulties.

Based on the above inclusion/exclusion criteria, the following 482 children were included: 69 children with ASD (mean age 13.78 (3.09) years; 49 (71%) males), 142 children with ASD and co-occurring ADHD (ASD/ADHD group; mean age 13.12 (3.31) years; 89 (62.7%) males), and 271 children with ADHD (mean age 12.63 (3.26) years; 172 (63.5%) males). The ADHD group included children with ADHD from a previous QbTest study [33]. There was no statistically significant difference regarding gender across groups, (χ^2^ (*df*) = 1.60 (2), *p* = 0.45), but a statistically significant difference was found regarding age distribution, (*F* (*df*) = 3.74 (2), *p* = 0.02). Among the children in both groups (ASD and ASD/ADHD) who had ASD, 72 (34.1%) had been previously diagnosed with Asperger’s disorder or high functioning autism, 15 (7.1%) with a pervasive developmental disorder (PDD) not otherwise specified (NOS), and 124 (58.8) with autism. In addition, 55 (26.1%) had some speech/language, learning, and/or motor difficulties. In the present study, we equated autism, Asperger’s syndrome, and PDD NOS with ASD. Among children with ASD, three (4.3%) received psychotropic medications other than MPH, while among children with ASD/ADHD, nine (6.3%) were drug-naïve, two (1.4%) received psychotropic medications other than MPH, and six (4.2%) were already treated with stimulants. In the ADHD group: 26 (9.6%) children also had some speech/language, learning, and/or motor difficulties. In the ADHD group, 15 (5.5%) children were drug-naïve, six (2.2%) received psychotropic medications other than MPH, and 10 (3.7%) were already treated with stimulants. For the remainder of the whole sample, regardless of whether a diagnosis had been assigned, no data were available regarding previous or current psychopharmacological treatments. Irrespective of possible previous or current psychopharmacological treatments, QbTest scores before MPH given on the testing day were analyzed for the study's first aim.

In line with the second aim, separate analyses were conducted that included a subgroup of children with ASD, ASD/ADHD, or ADHD who underwent the QbTest assessment twice on the same day. Once the baseline testing had been completed, the children were given a single dose of MPH. Up to four hours after MPH intake, the children were again tested with the QbTest (i.e., post-MPH assessment). Data for 28 children with ASD, 95 with ASD/ADHD, and 220 with ADHD were available for both pre- and post-MPH assessments (Table 1). Considering only the cardinal QbTest parameters for the pre-MPH testing, children in the ASD group who received MPH had significantly higher scores for the QbInattention (*p* < 0.01), but not for the QbActivity (*p* = 0.05) and the QbImpulsivity (*p* = 0.09) compared with those not receiving MPH. For the ASD/ADHD and ADHD group, children receiving MPH had significantly higher scores for the QbActivity, QbImpulsivity, and QbInattention (*p* < 0.01) before MPH was given, compared with those not receiving MPH.

**Table 1:** Main characteristics of children with ASD, ASD/ADHD, and ADHD tested with MPH

	ASD, *n* = 28	ASD/ADHD *n* = 95	ADHD, *n* = 220	*p* value
Male/female, *n* (%)	17 (60.7)/11(39.3)	56 (58.9)/39 (41.1)	134 (60.9)/86 (39.1)	0.95
Age, *M* (*SD*) years	14.63 (2.52)	12.99 (3.38)	12.55 (3.29)	< 0.01
MPH dose given, *M* (*SD*) mg	34.93 (7.33)	31.36 (13.26)	30.55 (10.12)	0.13
Time passed after MPH, *M* (*SD*) minutes	105.18 (34.92)	97.68 (29.04)	93.30 (24.03)	0.05
MPH formulation given, *n* (%)				0.12
Short acting	0	1 (1.1)	11 (5.0)	
Long acting	24 (85.7)	74 (77.8)	181 (82.3)	
Unknown	4 (14.3)	20 (21.1)	28 (12.7)	

*ASD* autism spectrum disorder, *ADHD* attention deficit hyperactivity disorder, *ASD/ADHD* ASD and co-occurring ADHD, *MPH* methylphenidate, *M* mean, *SD* standard deviation

### Instrument

The QbTest is a combination of a CPT and a motion-tracking system [38]. During a CPT task, a high-resolution infrared camera monitors participant’s head movements as they respond to stimuli appearing on a computer screen. The QbTest program summarizes the participant’s scores after completing the test in the form of raw scores and Q-scores, which are calculated using the normative data matched for gender and age. There are two versions of the test; one for children aged six to 12 years lasting 15 min and one for adolescents/adults between 12 to 60 years lasting 20 min. The children and adolescents in this study completed the version, corresponding to their age. Three cardinal parameters, QbInattention, QbActivity, and QbImpulsivity, provide a summary index of the CPT and motion-capture data. QbActivity includes data measured by the motion-capture device based on time active (in percentage [%]), distance (in meters [m]), area (in centimeters [cm^2^]), and micro-events (i.e., a number times the marker for the motion-tracking changes its position more than one millimeter), while QbInattention and QbImpulsivity includes CPT data, like omission errors (i.e., not responding to a target; [%]), reaction time and reaction time variation (in milliseconds [ms]), or commission errors (i.e., responding to a non-target; [%]). However, not all parameters have the same weight in these cardinal parameters (for details, see [38]). In this study, we focused on the main parameters for CPT and motor activity, error rate (i.e., the combined percentage of omission and commission errors compared to the total number of stimuli [%]), and the cardinal QbTest parameters (QbInattention, QbActivity, and QbImpulsivity) [38]. The cardinal parameters were derived from factor analysis, and the results were standardized to Q-scores (i.e., transformation of a skewed statistical distribution to normally distributed *z*-scores, where the mean equals 0 and the standard deviation equals 1) [38, 41]. Previous psychometric studies demonstrated good measurement properties for the QbTest, including good test–retest reliability [45]. Detailed information on the QbTest and a description of the norm database can be found in the QbTest technical manual [38].

### Statistical analyses

An analysis of variance (ANOVA) model, with age adjustment due to the significant differences in the distributions, was performed to test differences in QbTest scores between the groups (i.e., ASD vs. ASD/ADHD vs. ADHD). The difference in QbTest parameters between pre- and post-MPH was analyzed with a paired *t*-test for the ASD, ASD/ADHD, and ADHD groups separately. The Cohen’s *d* effect size was calculated, and its values were interpreted as small (< 0.5), medium (0.5–0.8), or large (> 0.8) [46]. In addition, an ANOVA (with Bonferroni correction), adjusted for age, was used for group comparisons regarding the differences in QbTest scores from pre- to post-MPH (i.e., the magnitude of change) across the groups. The level of significance was set at *p* < 0.05.

## Results

The mean (standard deviation [*SD*]) values for the QbTest parameters of the three groups are presented in Table 2. Statistically significant differences were found among the groups for all QbTest parameters. Four out of 12 QbTest scores were not statistically different between the ASD and ASD/ADHD group, while nine out of 12 between the ASD/ADHD and ADHD. Scores for reaction time and reaction time variation were similar for the ASD and ASD/ADHD group, but significantly lower compared to the ADHD group. Regarding omission errors, time active, distance, area, and micro-events the ASD/ADHD and ADHD groups had similar scores, and significantly higher scores than the ASD group. Scores for commission errors were similar across the three groups. Error rates were significantly higher in the ADHD than ASD group. The cardinal parameter QbInattention, was significantly different between the groups (*p* > 0.05); the ASD group had the lowest, and the ADHD group the highest scores. The ASD/ADHD and the ADHD groups had similar QbActivity and QbImpulsivity scores and significantly higher than the ASD group.

**Table 2:** QbTest mean (*SD*) scores for the three groups

QbTest parameter	ASD	ASD/ADHD	ADHD	Comparison^a^
Reaction time	532.91 (121.36)	546.57 (119.83)	575.33 (132.30)	ADHD > (ASD = ASD/ADHD)
Reaction time variation	171.33 (75.61)	193.38 (66.31)	222.69 (85.03)	ADHD > (ASD = ASD/ADHD)
Omission errors	10.30 (13.73)	17.88 (18.99)	21.55 (18.79)	(ASD/ADHD = ADHD) > ASD
Commission errors	5.54 (9.62)	9.09 (10.55)	11.09 (13.17)	ASD = ASD/ADHD = ADHD
Error rate	6.87 (10.89)	11.72 (10.34)	15.36 (14.17)	ASD = ASD/ADHD; ASD/ADHD = ADHD; ADHD > ASD
Time active	13.20 (14.77)	25.90 (22.11)	32.12 (24.20)	(ASD/ADHD = ADHD) > ASD
Distance	6.15 (4.78)	11.42 (9.93)	13.82 (11.37)	(ASD/ADHD = ADHD) > ASD
Area	22.64 (23.65)	47.35 (44.40)	56.92 (44.77)	(ASD/ADHD = ADHD) > ASD
Micro-events	3720.18 (2918.25)	6672.15 (4985.55)	7942.93 (5369.68)	(ASD/ADHD = ADHD) > ASD
QbInattention	0.45 (1.37)	1.00 (1.25)	1.51 (1.27)	ADHD > ASD/ADHD > ASD
QbImpulsivity	-0.26 (1.17)	0.52 (1.25)	0.45 (1.29)	(ASD/ADHD = ADHD) > ASD
QbActivity	-0.47 (1.24)	.52 (1.28)	0.75 (1.25)	(ASD/ADHD = ADHD) > ASD

^a^ANOVA, corrected for age, pairwise comparisons of the groups, *p* < 0.05; *ASD* autism spectrum disorder, *ADHD* attention deficit hyperactivity disorder, *ASD/ADHD* ASD and co-occurring ADHD

The mean (SD) values for the QbTest scores before and after given MPH (pre-/post-MPH) are presented in Table 3. After being given MPH, scores for reaction time, reaction time variation, omission errors, error rate, time active, distance, area, micro-events, QbActivity, and QbInattention in the ASD group decreased significantly, and scores for QbImpulsivity increased significantly. In contrast, scores for commission errors changed minimally (*p* = 0.57). Cohen’s *d* effect sizes were small to large (see Table 3). All scores for the ASD/ADHD and ADHD groups decreased significantly at the post-MPH assessment, with the effect sizes ranging between moderate and large for all but commission errors and QbImpulsivity, with small effect sizes. There were no statistically significant differences among the three groups for the magnitude of scores’, decrease from pre- to post-MPH in scores for reaction time, reaction time variation, commission errors, time active, and distance. The decrease in scores for omission errors and area was similar in the ASD/ADHD and ADHD groups, and these declines were more prominent than in the ASD group. In contrast, the decrease in error rate scores was more pronounced in the ADHD than in the ASD group, but not compared to the ASD/ADHD group. For micro-events, the decrease was also similar in the ASD/ADHD and ADHD group, which in turn was greater compared to the ASD group. The QbInattention scores decreased similarly in all three groups. The decrease of QbImpulsivity scores was similar in the ASD/ADHD and the ADHD groups. A marked increase of the QbImpulsivity scores was seen in the ASD group compared to the other two groups. The QbActivity scores decreased similarly in the ASD/ADHD and the ADHD groups, similarly in the ASD and ASD/ADHD group, but largely in the ADHD compared to the ASD group.

**Table 3:** QbTest mean (*SD*) scores before and after given MPH (pre-/post-MPH)

QbTest parameter	ASD			ASD/ADHD			ADHD			Comparison^a^
	Pre-MPH	Post-MPH	*p*, *d*	Pre-MPH	Post-MPH	*p*, *d*	Pre-MPH	Post-MPH	*p*, *d*	
Reaction time	597.92 (148.23)	493.61 (117.34)	< 0.01, 0.93	574.16 (119.01)	487.84 (108.44)	< 0.01, 0.96	583.16 (132.09)	488.06 (100.62)	< 0.01, 0.95	ASD = ASD/ADHD = ADHD
Reaction time variation	209.56 (88.88)	164.32 (90.97)	< 0.01, 0.92	213.14 (61.99)	158.75 (64.77)	< 0.01, 0.92	228.46 (82.53)	163.82 (80.57)	< 0.01, 0.91	ASD = ASD/ADHD = ADHD
Omission errors	16.33 (15.68)	11.23 (13.49)	0.01, 0.62	19.56 (16.18)	7.26 (10.33)	< 0.01, 0.99	22.63 (18.03)	8.42 (12.61)	< 0.01, 0.99	(ASD/ADHD = ADHD) > ASD
Commission errors	7.94 (13.48)	7.30 (12.98)	0.57, 0.11	10.24 (11.43)	7.25 (9.37)	< 0.01, 0.43	11.64 (13.47)	7.77 (10.34)	< 0.01, 0.46	ASD = ASD/ADHD = ADHD
Error rate	10.31 (14.26)	8.55 (14.20)	0.02, 0.49	13.05 (10.35)	7.08 (8.49)	< 0.01, 0.92	15.94 (13.79)	8.79 (13.01)	< 0.01, 0.75	ASD = ASD/ADHD ASD/ADHD = ADHD; ADHD > ASD
Time active	14.54 (14.35)	8.38 (8.81)	0.02, 0.52	30.33 (22.24)	17.61 (19.78)	< 0.01, 0.90	33.73 (24.19)	20.11 (24.34)	< 0.01, 0.77	ASD = ASD/ADHD = ADHD
Distance	6.85 (4.73)	4.99 (2.73)	0.04, 0.44	13.03 (9.74)	7.72 (8.05)	< 0.01, 0.75	14.54 (11.82)	9.08 (10.69)	< 0.01, 0.70	ASD = ASD/ADHD = ADHD
Area	26.13 (25.11)	15.21 (13.92)	0.04, 0.45	56.32 (44.25)	27.97 (35.97)	< 0.01, 0.89	59.82 (45.49)	31.77 (39.13)	< 0.01, 0.87	(ASD/ADHD = ADHD) > ASD
Micro-events	4086.92 (2874.15)	3013.17 (19.12.02)	0.03, 0.46	7632.12 (4965.94)	4720.31 (4141.68)	< 0.01, 0.89	8294.59 (5437.94)	5350.15 (5156.85)	< 0.01, 0.79	ASD = ASD/ADHD ASD/ADHD = ADHD; ADHD > ASD
QbInattention	1.06 (1.24)	-0.16 (1.25)	< 0.01, 1.18	1.30 (1.15)	-0.23 (1.27)	< 0.01, 1.47	1.62 (1.26)	-0.05 (1.35)	< 0.01, 1.39	ASD = ASD/ADHD = ADHD
QbImpulsivity	0.01 (1.41)	0.33 (1.48)	0.04, -0.43	0.72 (1.19)	0.29 (1.31)	< 0.01, 0.37	0.55 (1.29)	0.13 (1.21)	< 0.01, 0.36	ASD > (ASD/ADHD = ADHD)
QbActivity	-0.18 (1.15)	-0.71 (0.96)	0.02, 0.51	0.88 (1.13)	-0.19 (1.17)	< 0.01, 1.20	0.85 (1.20)	-0.31 (1.31)	< 0.01, 1.02	ASD = ASD/ADHD ASD/ADHD = ADHD; ADHD > ASD

^a^ANOVA corrected for age, pairwise comparisons of the groups, *p* < 0.05; *ASD* autism spectrum disorder, *ADHD* attention deficit hyperactivity disorder, *ASD/ADHD* ASD and co-occurring ADHD; *p – p* value for t test, *d* – Cohen’s d effect size

## Discussion

In the present study was evaluated the QbTest performance among children with ASD and co-occurring ADHD, as compared to those with ASD or with ADHD alone, and the effects of single-dose MPH on that performance was also assessed.

Considering the QbTest parameters for attention, children in the ASD group and in the ASD/ADHD group had similar reaction times and reaction time variations, but lower compared to the children in the ADHD group. At the same time, those with ASD/ADHD exhibited more omission errors compared to those with ASD, but similar compared to the ADHD group. The QbInattention data, revealed that children with ASD/ADHD had more overall attention deficits than those with ASD alone, and fewer than those with ADHD alone. Several previous studies reported that children with ASD/ADHD make more omission errors and exhibit increased reaction time variability than children with ASD and/or controls [20, 22, 23, 47–49]. One previous study found similar omission errors and variability in responses when comparing children with ASD, ADHD, and ASD/ADHD [50]. Another study showed that increased response time variability, but not commission errors, was found to explain the co-occurrence of autistic traits in ADHD [51].

Considering the QbTest parameters for impulsivity, children with ASD/ADHD had similar numbers of commission errors as the children with ASD and ADHD, respectively. QbImpulsivity scores, were similar between children with ASD/ADHD and ADHD, but higher than among the children with ASD. Findings from previous studies are partially consistent with ours; two papers reported that children with ASD/ADHD had similar commission errors compared to typically developing youths [49, 52], while other studies found differences when comparing ASD and ADHD regarding this parameter [20, 22, 50]. Another study showed that commission errors could not relate autistic traits and ADHD [51]. An important finding in our study was related to the error rates. Children with ASD/ADHD had similar error rates as children with ASD or ADHD. Contrary, previous studies reported that children with ASD/ADHD may have a greater propensity to make errors than children with ASD (e.g., [22, 23, 27]). In our study, however, there were significant differences between ASD and ADHD. Another important CPT parameter, omission errors (i.e., not responding to a target as a measure of selective attention), should be pointed out. Taking all findings from our study and consistent previous (e.g., [20, 22, 23]), omission errors could be a possible QbTest marker to differentiate ASD with attentional deficits from ASD alone.

Regarding the QbTest parameters for activity, children with ASD/ADHD had more atypical scores than children with ASD. Still, scores were poor as were for children with ADHD. This pattern implies that children with ASD/ADHD were more hyperactive than children with ASD, but similarly to those with ADHD. In this regard, hyperactivity traits as quantified by the QbTest, could be a marker to differentiate ASD with co-occurring ADHD from ASD alone. It has previously been suggested that QbTest could be used to differentiate children with ADHD from their unaffected siblings or unrelated controls [43].

After a single dose of MPH, children with ASD/ADHD and children with ADHD improved on all QbTest parameters, but the improvement had small clinical relevance for measures of impulsivity (i.e., commission errors and the QbImpulsivity). The children with ASD, on the other hand, did not significantly improve in commission errors and deteriorated significantly in the QbImpulsivity parameter, although simultaneously improving clinically relevant on the other QbTest parameters. Comparing how much the pre-test scores changed after the administration of a single dose of MPH, there was a similar improvement regarding deficits in two measures of attention (i.e., reaction time and reaction time variability, but not on omission errors), as well as the QbInattention parameter among the children with ASD, ASD/ADHD, and ADHD. Therefore, a single-dose MPH does likely improve attention deficits regardless of the disorder. Commission errors, as an impulsivity parameter, also improved similarly across the groups. However, the QbImpulsivity parameter showed deterioration in children with ASD. Conversely, this parameter exhibited similar improvements in the children with ASD/ADHD and ADHD after a single dose of MPH. We have previously observed that a single MPH dose can worsen impulsivity, especially in adolescents with ADHD [33]. Peled, Cassuto, and Berger [37] showed that after a single dose of 10 mg of MPH, processing speed improved in children with combined ASD and ADHD but had no effect on omission and commission errors. A possible explanation for this observed difference from our study could be that children were tested with a higher, on average 30 mg, the dose of MPH in the present study. However, our results in children with ASD/ADHD align with those of the CPT performance study by Pearson et al. [36], showing improved sustained attention, selective attention, and impulsivity/inhibition. Finally, four of the five activity parameters, including the QbActivity, improved similarly in children with ASD/ADHD as if in ASD and ADHD. Thus, if focusing only on ASD with co-occurring ADHD, it is indicative that a single dose of MPH markedly improved deficits in attention and substantially decreased motor activity levels while improved impulsivity to a small degree. In this regard, differences in the QbTest parameters before and after MPH indicate that the testing dose may be helpful in the prediction of the potential responses to treatment in children with ASD and ADHD and may increase the likelihood of recognizing individuals who could benefit from ongoing MPH treatment [53].

The present study has several limitations. First, the study was based on chart reviews and the reliability of the data could be questioned. Second, it was not available how it was decided to perform the QbTest, irrespective of later being assigned any neurodevelopmental diagnosis, behavioral, or mental disorder to a child. This is particularly relevant considering that those who received MPH had more atypical QbTest scores, and in the ASD/ADHD group more children had predominant attention deficits than in the ADHD group, which could bias the results. Third, the diagnostic procedures at the clinics have likely changed over time and diagnostic criteria used. Since this was a retrospective study using clinical data, one must expect that the clinicians may not have been blinded to the results of the QbTest. Accordingly, their diagnostic and therapeutic decisions may have been influenced, which imposes the possibility of bias. However, the fact that the results represent a non-selected clinical sample is the study's strength. Fourth, the data on concurrent psychopharmacological treatments at the time of assessment was incomplete, and only a small number of subjects could be classified as drug-naïve. Different doses of MPH were given during the QbTest, without communicating the rationale for this decision. Fifth, intellectual functioning can affect CPT performance [20], and therefore intellectual disability was an exclusion criterion in the present study. However, data on levels of intellectual functioning for the included children were not available for the analyses in the study. Finally, no additional measures or data were available which could be used to explain the variances in QbTest scores observed in the groups.

## Conclusions

Children with ASD and co-occurring ADHD exhibited more atypical QbTest performances than children with ASD. However, most of their QbTest patterns were similar to those observed in children with ADHD. Regarding the cardinal QbTest parameters, children with ASD/ADHD had more attention deficits, higher activity levels, and greater impulsivity than children with ASD. Children with ASD/ADHD had lower attention deficits but similar activity and impulsivity levels compared with those with ADHD. A single dose of MPH mitigated the deficits in attention in children with ASD/ADHD, in line with the children in the ASD and ADHD groups. MPH also improved impulsivity and decreased motor activity similarly in the ASD/ADHD and the ADHD groups. The results of our study indicate that combining a CPT with motion analysis could help differentiate ASD and ASD with ADHD in clinical practice and could provide additional information on immediate responses to MPH in these children. Considering the study's limitations, our results call for further prospective studies to confirm the role of the QbTest in the diagnostic process in children with symptoms of ASD and ADHD and during the evaluation of MPH response.

## Data Availability

All data generated or analyzed during this study are included in this published article.

## Abbreviations

ADHD: Attention deficit hyperactivity disorder
ASD: Autism spectrum disorder
CPT: Continuous performance task
FDA: Food and Drug Administration
MPH: Methylphenidate
QbTest: The Quantified behavior Test
SD: Standard deviation
